# Parkinson Disease Detection from Speech Articulation Neuromechanics

**DOI:** 10.3389/fninf.2017.00056

**Published:** 2017-08-25

**Authors:** Pedro Gómez-Vilda, Jiri Mekyska, José M. Ferrández, Daniel Palacios-Alonso, Andrés Gómez-Rodellar, Victoria Rodellar-Biarge, Zoltan Galaz, Zdenek Smekal, Ilona Eliasova, Milena Kostalova, Irena Rektorova

**Affiliations:** ^1^NeuVox Lab, Biomedical Technology Center, Universidad Politécnica de Madrid Madrid, Spain; ^2^Department of Telecommunications, Brno University of Technology Brno, Czechia; ^3^Department of Electronics, Computer Technology and Projects, Universidad Politécnica de Cartagena Cartagena, Spain; ^4^First Department of Neurology, Faculty of Medicine and St. Anne's University Hospital, Masaryk University Brno, Czechia; ^5^Applied Neuroscience Research Group, Central European Institute of Technology, CEITEC, Masaryk University Brno, Czechia; ^6^Department of Neurology, Faculty Hospital and Masaryk University Brno, Czechia

**Keywords:** neurologic disease, Parkinson disease, speech neuromotor activity, aging voice, hypokinetic dysarthria, random least squares feed-forward networks

## Abstract

**Aim:** The research described is intended to give a description of articulation dynamics as a correlate of the kinematic behavior of the jaw-tongue biomechanical system, encoded as a probability distribution of an absolute joint velocity. This distribution may be used in detecting and grading speech from patients affected by neurodegenerative illnesses, as Parkinson Disease.

**Hypothesis:** The work hypothesis is that the probability density function of the absolute joint velocity includes information on the stability of phonation when applied to sustained vowels, as well as on fluency if applied to connected speech.

**Methods:** A dataset of sustained vowels recorded from Parkinson Disease patients is contrasted with similar recordings from normative subjects. The probability distribution of the absolute kinematic velocity of the jaw-tongue system is extracted from each utterance. A Random Least Squares Feed-Forward Network (RLSFN) has been used as a binary classifier working on the pathological and normative datasets in a leave-one-out strategy. Monte Carlo simulations have been conducted to estimate the influence of the stochastic nature of the classifier. Two datasets for each gender were tested (males and females) including 26 normative and 53 pathological subjects in the male set, and 25 normative and 38 pathological in the female set.

**Results:** Male and female data subsets were tested in single runs, yielding equal error rates under 0.6% (Accuracy over 99.4%). Due to the stochastic nature of each experiment, Monte Carlo runs were conducted to test the reliability of the methodology. The average detection results after 200 Montecarlo runs of a 200 hyperplane hidden layer RLSFN are given in terms of Sensitivity (males: 0.9946, females: 0.9942), Specificity (males: 0.9944, females: 0.9941) and Accuracy (males: 0.9945, females: 0.9942). The area under the ROC curve is 0.9947 (males) and 0.9945 (females). The equal error rate is 0.0054 (males) and 0.0057 (females).

**Conclusions:** The proposed methodology avails that the use of highly normalized descriptors as the probability distribution of kinematic variables of vowel articulation stability, which has some interesting properties in terms of information theory, boosts the potential of simple yet powerful classifiers in producing quite acceptable detection results in Parkinson Disease.

## Introduction

Shaking palsy, as was first defined by James Parkinson in 1817 (Parkinson, 2002) or Parkinson Disease (PD) as is modernly known, is a pathology of neuromotor origin due to the decay of the neurotransmitter *dopamine* by degeneration of *substantia nigra pars compacta* in midbrain, which is characterized by bradikinesia, rigidity, resting tremor, freezing of gait and facial mask, and speech disorders, among other disease manifestations (Yunusova et al., 2008; Brabenec et al., 2017). Its effects in speech and phonation, gathered under the general term of hypokinetic dysarthria are quite relevant and notorious, to the point that they may become clear subjective clinical indicators of neuromotor deterioration (Goetz et al., 2007), in the sense that there is “compelling evidence to suggest that speech can help quantify not only motor symptoms …but generalized diverse symptoms in PD” (Tsanas, 2012). Hypokinetic phonation is characterized by voice blocking, changes in energy and fundamental frequency of a specific low frequency (tremor, or pathologic vibrato), hypotonic (asthenic) phonation, etc. (Gómez et al., 2017). Hypokinetic dysarthria may appear itself as a reduction in magnitude and velocity of articulatory movements, besides showing inter-articulator timing disturbances. These manifestations seem to be the result of neuromotor disfunctions affecting to “individual or collective movements of articulators such as the jaw, tongue and lips” (Yunusova et al., 2008). Disturbances in the temporal coordination of articulatory motion of speakers of PD or Amyotrophic Lateral Sclerosis (ALS) are one of the early marks of these neuromotor pathologies. The role of the tongue, jaw and lower lip movements seem to be behind these dysartrhias. Acknowledging these facts, there have been different approaches to evaluate phonation and articulation by quantitative analysis. Regarding phonation, an early work by Gamboa et al. (1997) tried to compare voice production from patients before and after dopaminergic treatment estimating distortion features as jitter, shimmer and noise-harmonic energy ratios using a popular voice analysis tool (CSL, 2017). Phonation analysis is based on the stability of fundamental frequency, energy and distortion measurements as harmonics-noise ratios (see Mekyska et al., 2015 for a comprehensive review). Regarding articulation, quality analysis is based on acoustic measurements on the span defined by the first two formants, as the Vowel Space Area (VSA) and the Formant Centralization Ratio (FCR), defined by Sapir et al. (2010). Other approaches depend on measuring the articulation positions either from scan images (Bouchard et al., 2016) or from invasive methods using sensors attached to different parts of the oral cavity (Savariaux et al., 2017). The interest of acoustic analysis is based on its non-invasive nature and the immediacy of data gathering, as it uses speech recordings. Its weakness is due to the presence of certain ill-posed inversion problems which may jeopardize the uniqueness of the solutions. Nevertheless, if properly handled, these weaknesses may be overcome. One of the limitations of the VSA or FCR indices come from their static nature. Speakers are recorded uttering running speech, to obtain good estimates of extreme formant positions on the vowel triangle (VSA and FCR are evaluated on these estimations). Therefore, the text of the utterance should contain a rich representation of the extreme vowel repertoire; typically at least the vowels [a:], [i:], and [u:] should be present. The problem is that to be reliable and robust enough, VSA and FCR must depend on average formant positions, and once these have been established, a static photo-finish is obtained. Much of the interest of articulation analysis is not only in static formant representations (although these may reveal as very interesting data in diseases as ALS), but also in the dynamic behavior of articulation positions (Green, 2015), as these have been recognized as behavioral landmarks in hypokinetic dysarthria (Yunusova et al., 2011), in the belief that dynamic articulation descriptions may be more sensitive to this behavior than static estimations on the vowel triangle. In the present work research is oriented to define a measurement which collects articulation kinematics, opening the possibility of estimating articulation dysfunction from simple and short utterances, as sustained single vowels as [a:], easing data gathering and analysis. Therefore, the paper is structured as follows: section Methods and Materials is devoted to treat the methods proposed for the detection of PD speech by a description of hypokinetic dysarthria in terms of articulation kinematics (Section Kinematic description of speech articulation), explaining the fundamentals inspiring articulation biomechanics, giving an example contrasting the statistical properties of articulation kinematic distributions from a normative control and a PD female patient, to better understand the dynamic properties of the features proposed. The classification algorithms to be used are introduced in section Classifier proposed for PD speech detection, with special emphasis in their representation properties. Section Materials is dedicated to describe briefly the dataset used in the experiments using articulation features derived from vowel utterances. Section Results and discussion is devoted to present classification results from a single run, and due to the stochastic nature of the classification algorithm, to provide a statistical description of the optimal parameter settings and classification scores following Monte Carlo simulations. Section Conclusions briefly describes the most relevant conclusions derived from the study, as the high semantic value of articulation kinematics and the high efficiency of the stochastic feed forward networks in the proposed classification tasks.

## Methods and materials

### Kinematic description of speech articulation

**Figure 1:**
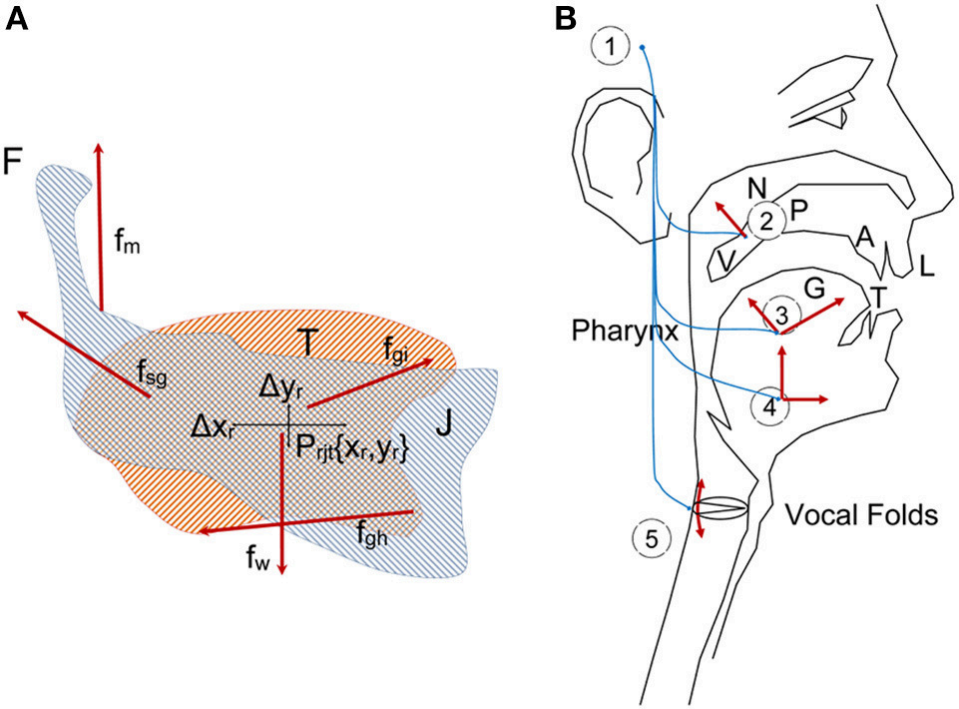
**(A)** Jaw-Tongue Biomechanical System. The jaw bone is represented in light gray, the tongue structure is represented in light orange. The point P_rjt_ given by {x_r_, y_r_} is the reference point of the biomechanical system. **(B)** Speech articulation neuromotor and biomechanical system.

Speech production involves cognitive and neuromotor resources of the human physiology, from planning and instantiation in the linguistic neuromotor cortex (Demonet et al., 2005), to muscle activation in the pharynx, tongue, larynx, facial mask, chest, and diaphragm through a wide network of neuromotor pathways. The intermediation between cortical neurons (primary) and neuromotor units activating the muscles takes place in the basal ganglia, where secondary neurons connected through sub-thalamic secondary pathways produce sequences of motor actions with intervention of other parts of the central nervous system, as the cerebellum, hippocampus and frontal lobes. It is a well-established fact that neurotransmission failures in the basal ganglia due to the progressive death of dopaminergic neurons in the substantia nigra pars compacta are the main reason behind PD neuromotor symptoms: “The substantia nigra is the origin of the nigrostriatal pathway, which travels to various structures within the basal ganglia…The dopamine deficiency in this nigrostriatal pathway and the basal ganglia account for most of the typical features of PD. Once the brain is no longer able to compensate for this dopamine loss, there are a number of effects which can occur. Typical symptoms include muscle rigidity, akinesia, bradykinesia, and tremor…” (Goberman and Coelho, 2002), more specifically “The essential neuropathological changes in PD are a loss of melanine-containing dopaminergic neurons in the substantia nigra pars compacta…This results in a dysfunction of the basal ganglia circuitries, which is an integral part of cortico-basal ganglia-cortical loops that mediate motor and cognitive functions (Harel et al., 2004). Besides affecting limb movements, neuromotor failures may affect all motor functions in the body, among them respiration (diaphragm, chest), phonation (larynx) and articulation (velopharyx, jaw, tongue, lips and other facial mask muscles). These failures result in dysphonic and dysarthric behavior manifested in speech as different acoustic correlates. As far as dysarthria is concerned, these manifestations are the result of improper work of the articulation biomechanics, which is mainly reflected in the action of the tongue, jaw and lower lip, as mentioned before. In what follows, a biomechanical system of the jaw-tongue will be proposed, which may be modeled to estimate the neuromotor behavior of the system, and provide specific markers of proper or improper neuromotor activity. For the purposes of the present study, only the Jaw-Tongue Biomechanical System (JTBS), as seen in Figure 1, is to be considered.

The jaw (J) is fixed to the skull bone at fulcrum (F) as in a third-class lever system. The tongue (T) is supported by jaw and the hyoid bone. The reference point of the jaw-tongue system *P*_*rjt*_ is defined at {*x*_*r*_*,y*_*r*_}, where forces acting on the system induce movements in the sagittal plane (*x*: horizontal, or rostral-caudal, *y*: vertical, or dorsal-ventral); these forces are *f*_*m*_ (masseter), *f*_*sg*_ (styloglossus), *f*_*gi*_ (intrinsic glossus), *f*_*gh*_ (hyoglossus), and *f*_*w*_ (gravity). The kinematic displacements experienced by *P*_*rjt*_ as a result of these forces are given as {Δ*x*_*r*_, Δ*y*_*r*_}. Lateral movements orthogonal to the sagittal plane are assumed small enough not to be considered (system with only two degrees of freedom). The functioning of the speech articulation neuromotor and biomechanical system may be explained in Figure 1B. The phonation and articulation systems are governed by specific neuromotor units activated from the bulbar structures in the midbrain (1), which control the retraction of the velopharyngeal switch (2) in nasalization, activate tongue movements up, down, back and forth (3: intrinsic and extrinsic lingual muscles), modify lower jaw position (4: masseter, stylo and hyoglossus), or control larynx and vocal fold configuration (5: vocalis, crico-arytenoid, transversal and oblique). In the present study, as far as PD neuromotor degeneration is considered, only subsystems (3) and (4) will be taken into account. Neurotransmission failures in the basal ganglia affect to fine movement control involving proprioceptive feedback. Depending on the structures involved (cerebellum, hippocampus, and frontal lobes…) different feedback loops with distinct latencies are active in motor control. These loops are continuously over or underactivating the neuromotor flow depending on the response of the sensory neurons, therefore a slow amplitude tremor may be present in the muscle activity in the band over 25 Hz due to fine tuning, which is considered a correlate of healthy motor behavior. The lack of dopamine due to degradation of dopamine-producing cells in *substantia nigra pars compacta* may lead to improper feedback loop function, and this produces insufficient or excessive excitatory flow (inducing hypo- or hypertonicity), or in a sloth overcorrecting flow, which results in wider muscle tension oscillations in the band from 5 to 8 Hz, and is considered a pathological tremor (Mertens et al., 2013). Therefore, hypo- hyper- or unstable muscle tone are markers of possible PD neurodegeneration, and these can be traced from speech. A feasible option to monitor dysarthria is to resource to acoustic correlates of articulation. It is a well established fact that muscles in the jaw-tongue structure may modify the vocal tract in a predictable and highly controllable way (Dromey et al., 2013), and acoustic phonetics provides a good description of the acoustic results summarized as the vowel polygon by IPA (2015) Figure 2.

**Figure 2:**
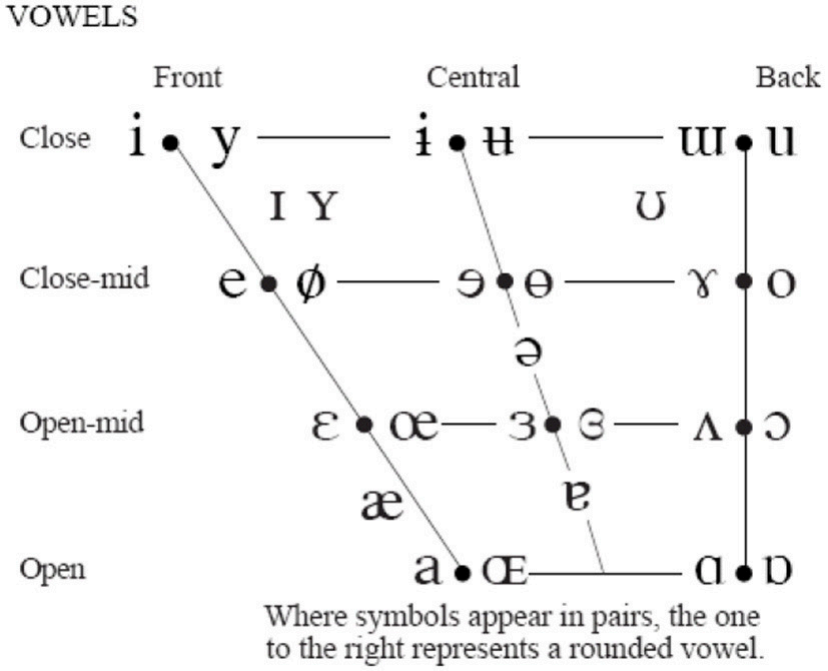
IPA vowel chart.

The IPA chart explains the association between articulation gestures (open-close in the vertical dimension and back-front in the horizontal one) and acoustic properties (formants) in a simple way, which may be more formally established (Sanguinetti et al., 1997). As vowel positions are related to formant descriptions, a possible way to estimate articulation dynamics could be through formant kinematics (Carmona et al., 2016). Let's assume that the first two formants {*f*_1_*, f*_2_} of a speech segment are known. A general, unspecific functional relationship could be established among formants and the reference point as

(1) $$\begin{array}{l} \left[\begin{array}{c} f_{1}\left(t\right)\\ f_{2}\left(t\right) \end{array}\right]=\left[\begin{array}{cc} a_{11} & a_{12}\\ a_{21} & a_{22} \end{array}\right]\left[\begin{array}{c} x\left(t\right)\\ y\left(t\right) \end{array}\right] \end{array}$$

where *a*_*ij*_ are the transformation weights explaining the position-formant association, and *t* is the time. This relationship is known to be one-to-many, i.e., the same pair of formants {*f*_1_*, f*_2_} may be associated to more than a single articulation position. This inconvenience may be handled by modeling the joint probability of all the possible articulation positions associated to a given formant pair (Dromey et al., 2013).

In the sequel the following important assumptions have been taken into account:

- The tongue top surface, in oposition to the teeth, alveolar ridge, palate and velum is the primary element configuring the main articulation cavity (Gerard et al., 2006).
- The transversal section normal to sound propagation of the main articulation cavity is inversely proportional to the tongue profile in the sagittal plane.
- The lower formants {*f*_1_*, f*_2_} are specifically determined by the concatenate tube equivalent model of the main articulation cavity.
- The kinematic displacements {Δ*x*_*r*_, Δ*y*_*r*_} are limited to millimeter oscillations around the reference point *P*_*rjt*_ (small signal hypothesis).
- The tongue profile in the sagittal plane is directly related to the kinematic displacements.
- Labialized sounds will not be considered.
- The system given by Equation (1) may be considered linear, time-invariant and invertible.

Under the above stated conditions, the system in Equation (1). may be inverted, as

(2) $$\begin{array}{l} \left[\begin{array}{c} x\left(t\right)\\ y\left(t\right) \end{array}\right]=\left[\begin{array}{cc} w_{11} & w_{12}\\ w_{21} & w_{22} \end{array}\right]\left[\begin{array}{c} f_{1}\left(t\right)\\ f_{2}\left(t\right) \end{array}\right] \end{array}$$

where *w*_*ij*_ are the weights of the inverse system. The algorithmic methodology implied in the process of deriving kinematic variables from acoustical ones depends on the estimation of the first time derivative of this system, associating formant derivatives in time with the reference point kinematics

(3) $$\begin{array}{l} \left[\begin{array}{c} v_{x}\left(t\right)\\ v_{y}\left(t\right) \end{array}\right]=\left[\begin{array}{cc} w_{11} & w_{12}\\ w_{21} & w_{22} \end{array}\right]\left[\begin{array}{c} \frac{df_{1}\left(t\right)}{dt}\\ \frac{df_{2}\left(t\right)}{dt} \end{array}\right] \end{array}$$

where it has been assumed that *v*_*x*_ and *v*_*y*_ are the caudal-rostral and dorsal-ventral velocities of the reference point. It may be hypothesized that the dorsal-ventral velocity will be mostly related to changes in the second formant (back-front), and that the caudal-rostral velocity will be related to the dynamics of the first formant (up-down). This is equivalent to consider that *w*_11_ and *w*_22_ will be negligible compared to *w*_12_ and *w*_21_.

Therefore, the absolute kinematic velocity (AKV) of the reference point may be estimated as

(4) $$\begin{array}{l} |v_{RP}\left(t\right)|={\left[{\left(w_{12}\frac{df_{2}\left(t\right)}{dt}\right)}^{2}+{\left(w_{21}\frac{df_{1}\left(t\right)}{dt}\right)}^{2}\right]}^{1/2} \end{array}$$

**Figure 3:**
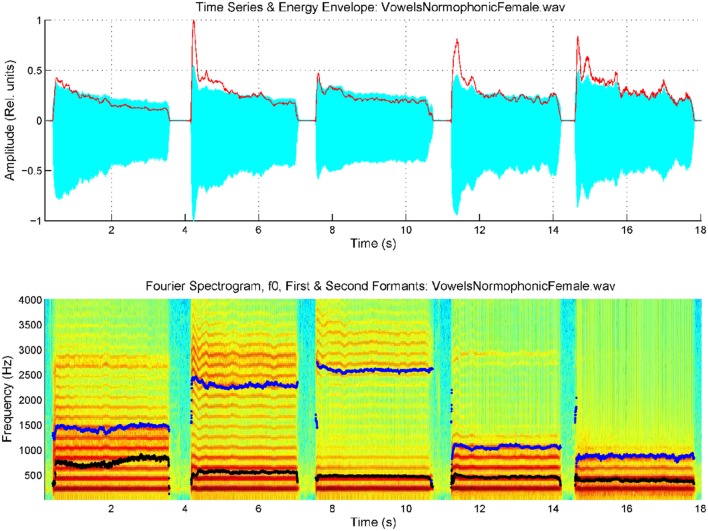
Spectrogram of an utterance with the five cardinal vowels [a:], [e:], [i:], [o:], [u:] by a female normative speaker, with the first two formants superimposed (*f*_1_: black, *f*_2_: blue). Top: speech trace and energy envelope (red). Bottom: spectrum and formants.

Reliable estimates for these scale factors were obtained from different calibration exercises, for example diphthong articulations involving changes in the positions of the reference point which show a fast and monotonous change, for instance in the repetition of the sequence /aiu/ uttered as […ajijuwa…]. The averaged estimations for both coefficients from such an utterance by a male speaker were found to be respectively *w*_12_ = 1.62.10^−3^ cm.s and *w*_21_ = 1.47.10^−3^ cm.s. The methodology to estimate the AKV in a practical case consists in evaluating the first two formants {*f*_1_*, f*_2_} by adaptive inversion of the speech segment after removing radiation effects to produce an estimate of the vocal tract transfer function in time and frequency. Formant positions are obtained either from the local maxima of the transfer function in each time instant, or from the polar positions of the prediction polynomials used to invert the speech segment. Formant estimates are used to evaluate the AKV, and its normalized histogram for the record segment being processed is to be defined as the non-parametric descriptor of the AKV statistical properties. In what follows a contrastive example showing the estimation of the AKV statistical distribution from its histogram is to be shown. The utterances of the five vowels [a:], [e:], [i:], [o:], and [u:] were recorded and used in the example. The five vowel record from a 34 year-old normative female speaker is given in Figure 3.

**Figure 4:**
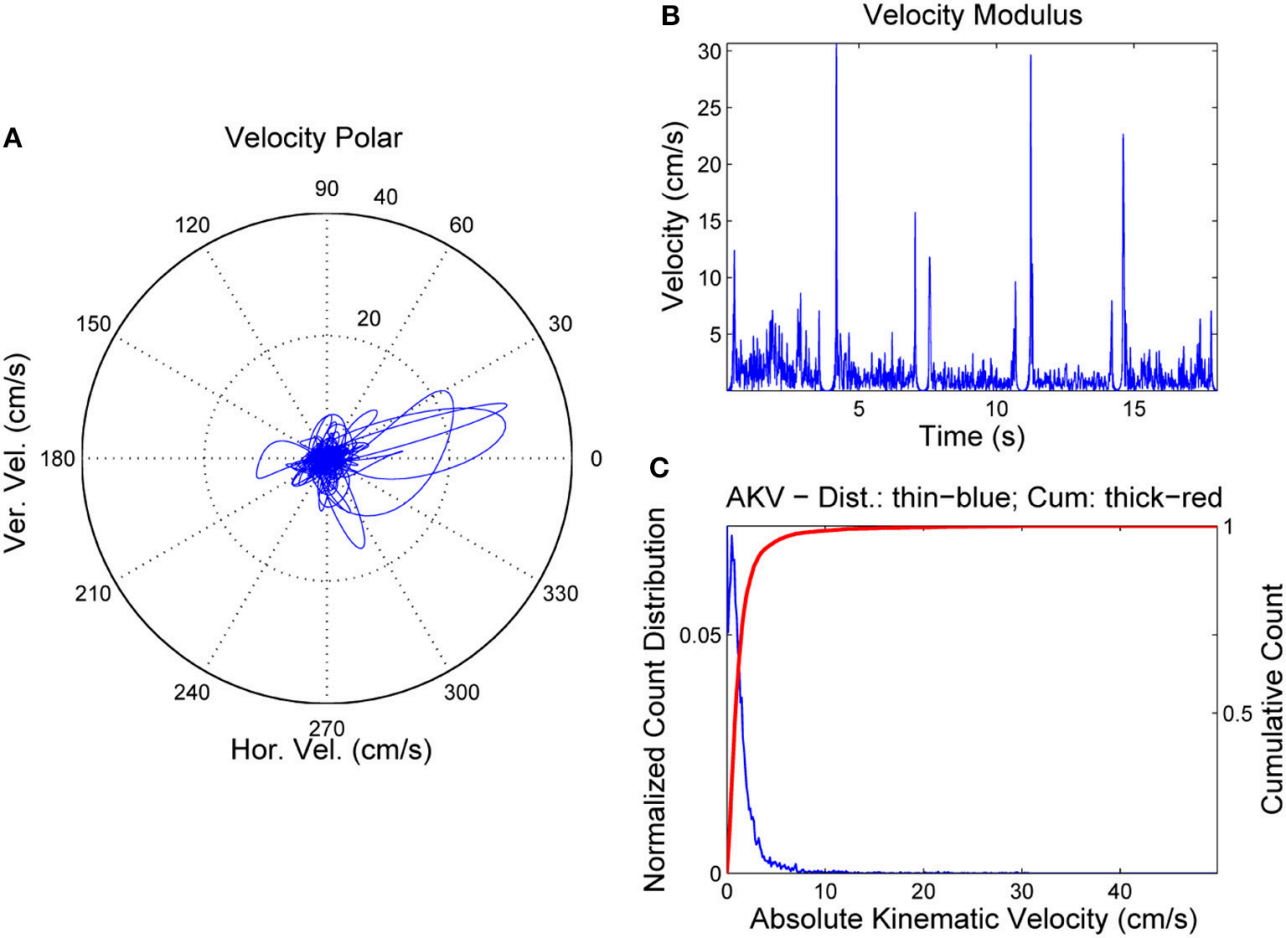
AKV from a normative female case. **(A)** Reference point velocity in module and angle. **(B)** AKV in the time domain. **(C)** AKV probability distribution (blue) and cummulative distribution (red).

The spectrogram was evaluated from a 9-order adaptive lattice-ladder filter to remove the influence of the glottal residual, sampled at 8 kHz and 16 bits (Haykin, 2013). Estimations of the spectrum using Linear Prediction Spectral Techniques were obtained each 2 ms. The next step will be to refine formant estimates to eliminate glitches and other hazards which could produce artifacts in evaluating kinematic variables. This is accomplished using a low-order predictive filter on each formant estimate. The kinematic variables *v*_*x*_ and *v*_*y*_ are evaluated from the derivatives of *f*_2_ and *f*_1_, as given by Equation (3). As formant kinematics is related with the neuromotor activity driving the jaw-tongue biomechanical system, frequency contents above 20 Hz are not pressumably of interest, due to the inertial properties of the system, therefore to estimate *v*_*x*_ and *v*_*y*_ formant derivatives have been low-pass filtered at a cutoff frequency of 20 Hz. Using these estimates, the AKV given in Equation (4) for the vowels in Figure 3 is shown in Figure 4.

**Figure 5:**
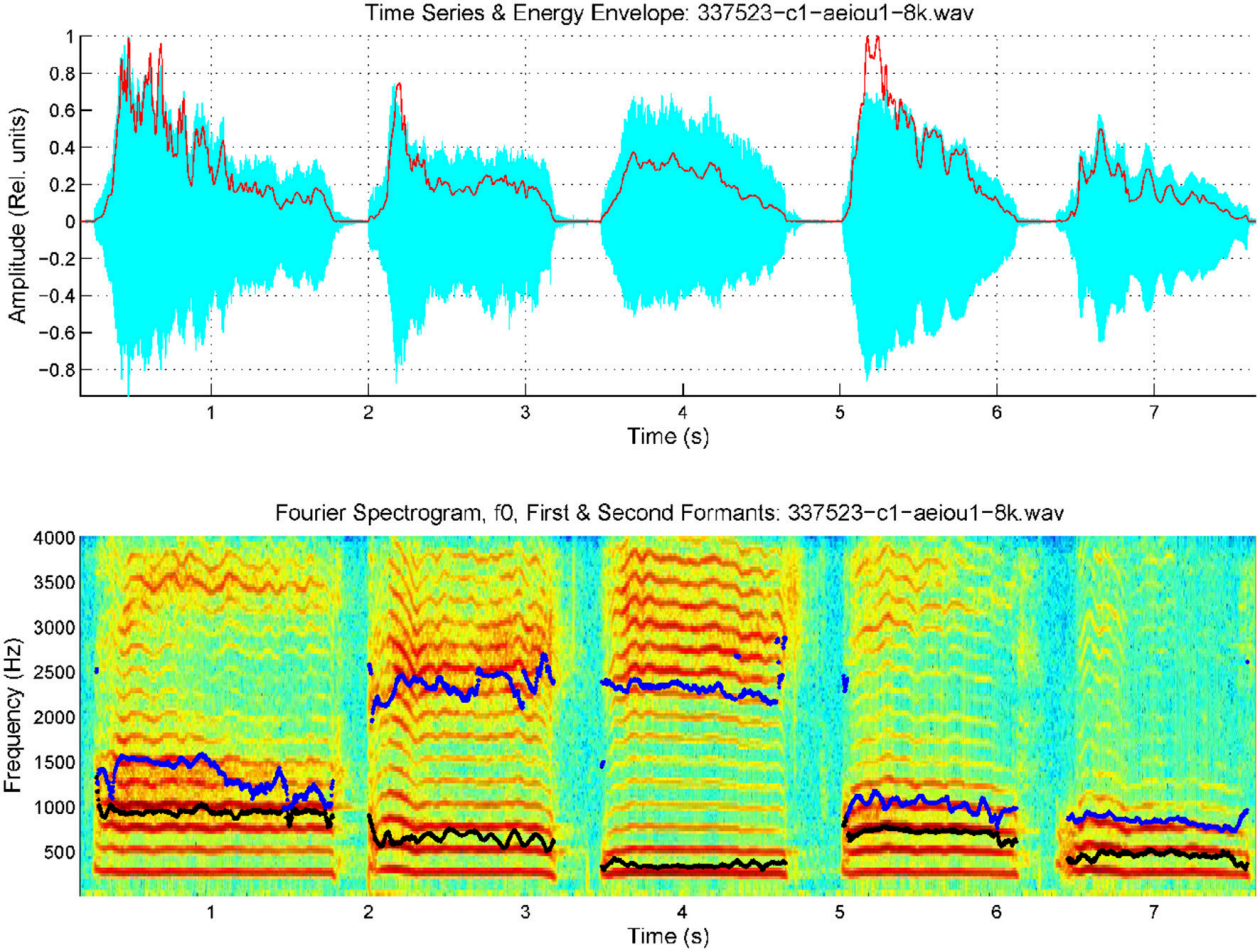
Spectrogram of an utterance with the five cardinal vowels [a:], [e:], [i:], [o:], [u:] by a female PD patient, with the first two formants superimposed (*f*_1_: black, *f*_2_: blue). Top: speech trace and energy envelope (red). Bottom: spectrum and formants.

In Figure 4A the reference point velocity is given in horizontal and vertical components describing a plot in module (AKV) and angle. The zero degree angle corresponds to the forward horizontal direction movement of the reference point (toward lips). In Figure 4B the AKV is presented the time-domain, showing large spikes in the vowel onsets and decays, and reflecting a much smaller activity in the vowel nuclei, supposedly due to the residual tremor amplitude induced by healthy neuromotor tuning. Figure 4C shows the AKV probability density function and its cummulative distribution, derived from the histogram of AKV values in amplitude. It may be seen that the probability density distribution (blue) shows a peak for low velocities, and a gentle decay toward 10 cm.s^−1^, which may be seen as an indicator of healthy behavior. The large loops in the right part of the polar plot in Figure 4A are related to large movements of the reference point due to adjustments of the jaw-tongue system in the vowel onsets, specially in [e:] (forwards) and [o:] (backwards), whereas the activity in the vowel nuclei is seen as a cloud of small amplitude actions in the center of the plot. The probability density function of the AKV will be used as a functional descriptor to detect articulation normality as explained in Section Materials. To stress the relevance of AKV in articulation stability detection, a similar analysis will be shown in Figure 5 from a 72 year-old female patient suffering from PD, grade 2 in Hoehn andYahr scale (Hoehn and Yahr, 1967).

**Figure 6:**
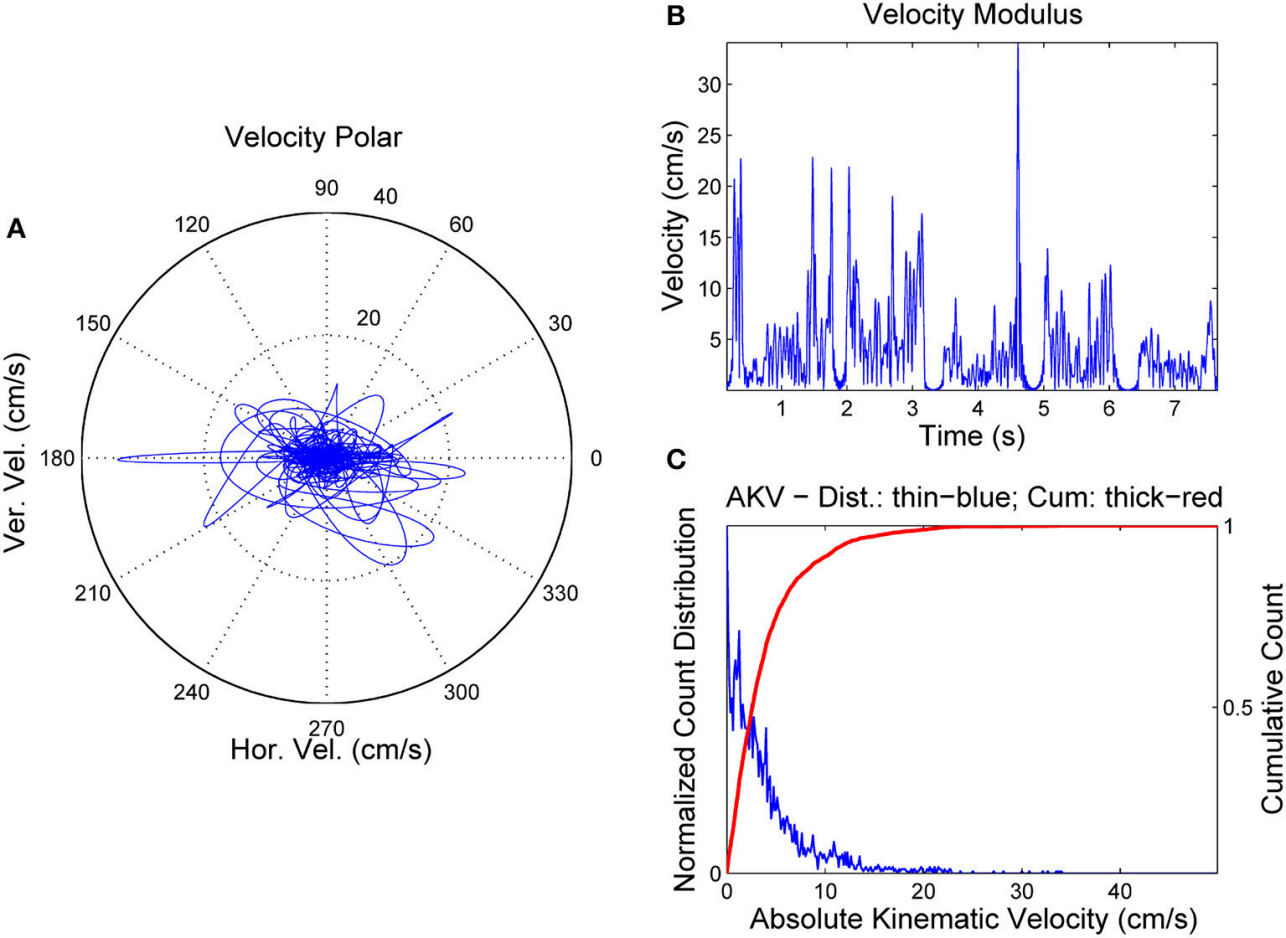
AKV from a PD female case. **(A)** Reference point velocity in module and angle. **(B)** AKV in the time domain. **(C)** AKV probability distribution (blue) and cummulative distribution (red).

Relevant tremor and articulation instability can be clearly appreciated in this case, both in harmonics as in formants. It must be said that these two tremors need not be correlated, as they depend on different neuromotor pathways, phonation being controlled by laryngeal nerves (vagus), whereas articulation depends mainly on jaw, lingual and facial nerves (branches of trigeminal), and PD may affect both systems differently. The statistical characterization of the AKV is given in Figure 6 as before.

It may be seen that horizontal and vertical velocities behave quite differently in the PD case than in the normative case. In the PD case the movements in the vowel onsets are significantly smaller, but much stronger in vowel nuclei, whereas their frequency is much smaller (around 8 Hz), which is a clear indication of pathological tremor being present. It may be seen also that the AKV horizontal and vertical loops move forwards and backwards as well as up and down, an indication of intense activity during vowel nuclei, as the speaker's proprioceptive system is trying to adjust vowel positions, but an improper feedback loop produces overshoot and undershoot in the articulation gestures, and this instability results in tremor. The statistical quality of articulation is captured in the AKV probability density function (Figure 6C) which shows activity over 10 cm.s^−1^, and the decay is slower. To evaluate the different statistical behavior of both AKV distributions non-parametric tests were conducted assessing the non-normal behavior of both distributions, as well as their independent origin. The test results are given in Table 1.

**Table 1:** Different behavior between the AKV distributions of the PD patient and the normative subject.

**Subject/Test**	**Test result**	**Test interpretation**
Normative female (model)–Lilliefors test	*p* < 0.001	Normality rejected
PD female patient (target)–Lilliefors test	*p* < 0.001	Normality rejected
PD vs. Normative–Kolmogorov-Smirnov	*p* = 2.44 10^−5^	Same distribution rejected
PD vs. Normative–Wilcoxon-Mann-Whitney	*p* = 0.0103	Same distribution rejected

It becomes evident that both AKV distributions do not pass the normality tests. Besides, both can be considered different either under the Kolmogorov-Smirnov and the Wilcoxon tests for a significance level of 0.05.

**Figure 7:**
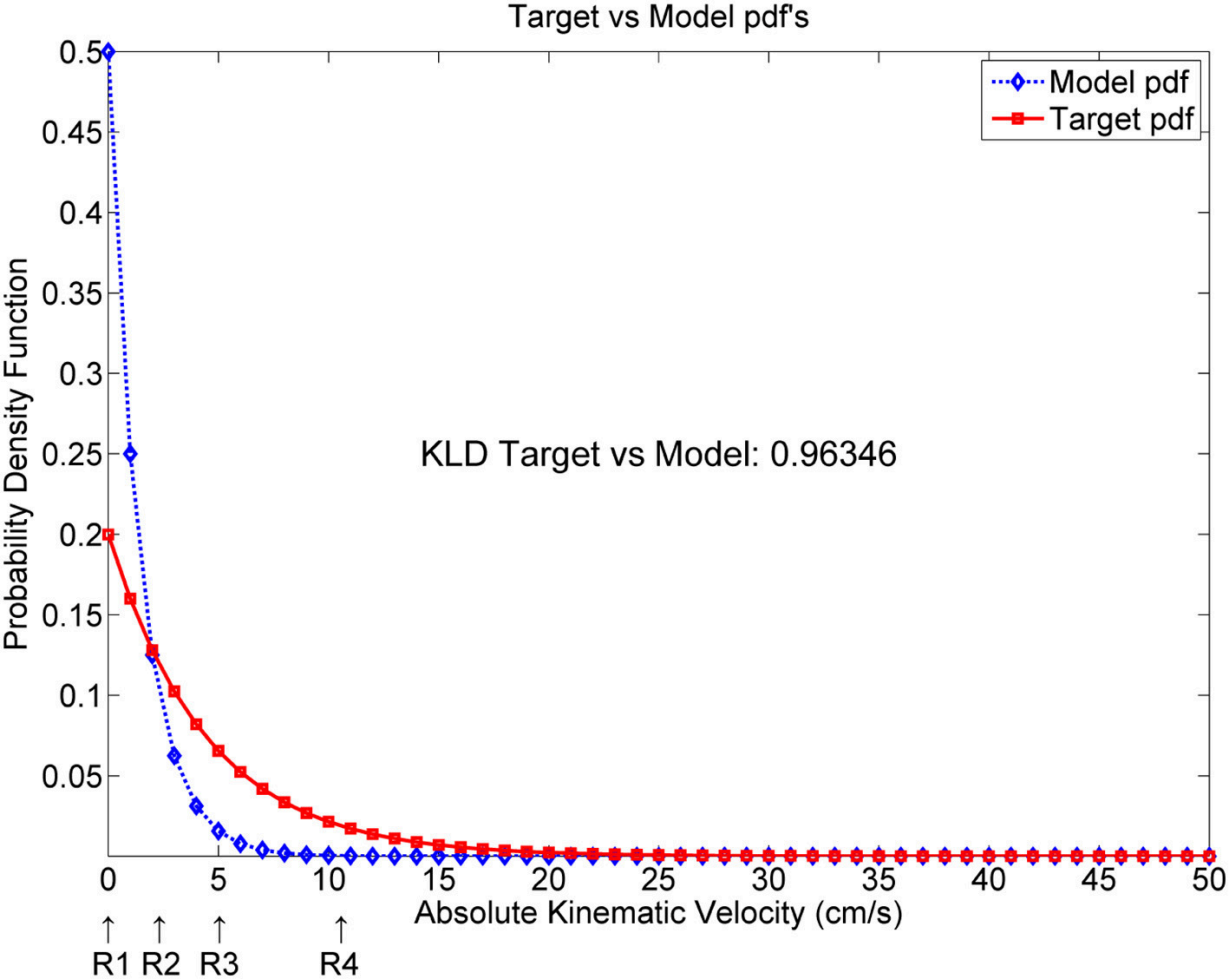
Simulated AKV probability density functions from a target (pathologic speaker) and a model (normative speaker). KLD: Kullback-Leibler Divergence.

At this point, it will become clear that the AKV probability density function may be a good candidate marker to establish differentiation between stable and unstable articulation induced by PD. Due to the nature of AKV as given in Equation (4) it is clear that its probability density function may be modeled as a χ^2^ (chi-square) distribution with two degrees of freedom (NIST, 2015), and its general shape will obey the pattern given in Figure 7, where the differential behavior of speech of PD patients with respect to healthy controls, following χ^2^ distributions is simulated as well. The AKV probability distribution from a healthy subject (diamond marks) is expected to follow a faster decay than the one from a PD patient when uttering sustained vowels. As the area under both distributions must equal one (being probability distributions), the control distribution is expected to start from a larger value than the pathological one and show a faster decay, confirmed by the results shown in Figures 4C, 6C. The difference between AKV probability densities can be established in terms of the Kullback-Leibler Divergence (KLD) following Information Theory principles (Cover and Thomas, 2006). For instance, the estimated KLD in the example of Figure 7 is 0.96346, as given in the plot. The kinematic behavior of a given utterance is encoded in its probability distribution, having into account that the AKV is a correlate of the horizontal and vertical movement speed of the jaw-tongue reference point, evaluated from a histogram of counts, in the following regions

R1. Silent intervals and pauses are accumulated as counts in the origin of the abscise point (zero bin). The higher this value, the larger the number of pauses, and the longer their duration.
R2. If formants remain stable or slightly changing during vowel nuclei the associated counts to these regions will be accumulated near the origin, under 3 cm.s^−1^.
R3. Smooth formant adjustments due to glides and approximants are to be found around 5 cm.s^−1^.
R4. Values of the AKV probability density function above 10 cm.s^−1^ are to be expected in vowel onsets following stop consonants and in other sharp phonation changes.

The way in which probability functions distribute on these four regions will depend on the kind of pathology being monitored, and on the type of utterance being analyzed. For instance, in pathologies were sentences are used to detect the residual articulation competence, as in Amyotrophic Lateral Sclerosis it will be expected that distributions from patients will show less activity in R3 and R4 than controls. In certain cognitive pathologies where fluency is compromised, it will be expected a larger number of counts in R1 relative to controls if continuous speech is monitored. In the case of PD patients uttering sustained vowels or sequences of vowels, the activity in R2, R3, and R4 is expected to be larger than in controls. This behavior will be used to monitor a database of sustained [a:] uttered by PD patients. In the next section these normalized distributions will be used in classification experiments.

### Classifier proposed for PD speech detection

**Figure 8:**
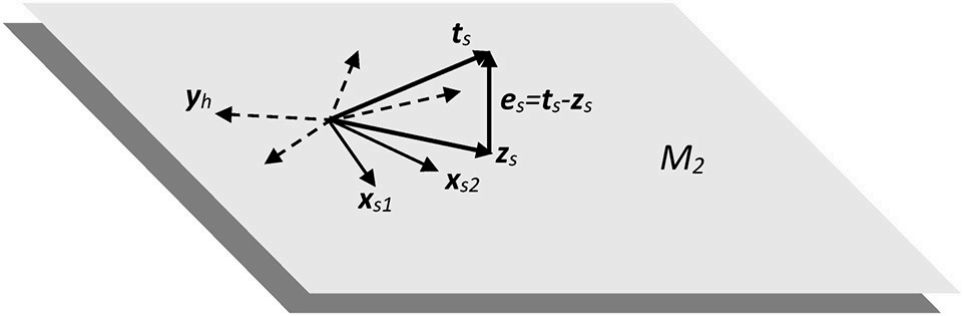
Subspace of two dimensions (M_2_) showing the dispersion and projection processes implicit in the RLSFN used in the experimental framework.

The classifier proposed is a specific kind of supervised multiple-layer artificial neural networks (ANN's) where the weights between input and output layers are fixed following stochastic methods (input layer) and least-squares learning bases (output layer), which will be referred as Random Least-Squares Feed-forward Networks (RLSFN), originally known as Random Vector Functional-Link Networks (RVFLN). These networks fulfill certain optimization criteria, but do not implement them in a successive sequence of weight adjustment iterations; on the contrary, weights are determined in specific fixed steps. They were proposed in 1988 by Broomhead and Lowe (1988), and in 1994 by Pao et al. (1994) with slightly different formulations. Later on, they were redefined by Huang and Siew (2004) under the name of extreme learning machines. They are especially well suited to solve n-class decision problems when the training set is composed of individual samples randomly selected from a given population. If the training set is derived from a temporal (stochastic) process in which samples cannot be considered independent in time, an adaptive formulation of weight adjustments should be used, in a mixed stochastic-adaptive methodology. Therefore, these RLSFN's can be considered within a family of “block” solutions, rather than to adaptive ones. Correspondingly, the results produced have to be validated under statistical terms as well. In the case under study the problem to be solved is that given a set of probability density feature vectors from speech samples by matrix ***X*** and their corresponding classification target marks given in matrix ***T*** (supervised training) specifying if sample vector ***x*** is to be associated by a target ***t*** as a member of a given class or category *C* (for instance, *C*_*n*_ for normative or *C*_*p*_ for pathological, in the present case), a mapping system has to be found based by linear projections and nonlinear kernels, to associate ***X*** to ***T*** minimizing an interclass confusion error function defined in statistical terms. This is a typical two-class problem as is the association of targets to two possible classes (normative or pathological), a two-layer RLSFN has been used. It will be assumed that the input ***x*** ϵ ${\mathbb{R}}^{n_{s}}$ is a row vector from an ***n***_*s*_ ***x n***_*f*_ matrix ***X*** ϵ ${\mathbb{R}}^{n_{s}\times n_{f}}$, each row *1* ≤ *i* ≤ *n*_*s*_ corresponding to a feature vector from the *i-th* subject, where *1* ≤ *j* ≤ *n*_*f*_ is the *j-th* feature index. Indeed, as feature vectors are probability density functions, in general *0* ≤ *x*_*ij*_ ≤ *1*. Target components will be defined as *t*_*s*_ ϵ {0,1}, 0 for normative, and 1 for pathologic samples. The first layer of the RLSFN will be defined as ***W***_1_ ϵ ${\mathbb{R}}^{n_{f}\times n_{h}}$, where *1* ≤ *k* ≤ *n*_*h*_ is the number of hyperplanes projecting input vectors **x** on the hidden nodes of the RLSFN, as by

(5) $$\begin{array}{l} Y=W_{1}X \end{array}$$

where ***W***_1_ is originally filled with real random values following a normal distribution with μ = 0 and σ = 1. This operation may be seen as building *n*_*h*_ linear combinations of the input vector ***x*** on an *n*_*s*_ dimensional subspace, where the weights of the linear combinations are random real numbers, therefore defining a set of *n*_*h*_ vectors ***y***. A reduced 2-dimensional example to visualize this construction is depicted in Figure 8.

In the 2-dimensional example shown in Figure 8, input samples are represented by vectors ***x***_*s*1_ and ***x***_*s*2_ from two different subjects (assuming a two-class detection example, as is the case with normative and pathologic subjects, for instance). The linear combinations implicit in expression Equiation (5). produce a set of randomly distributed vectors (in dash), of which **y**_*h*_ is an example, *h* being the reference to a specific distribution hyperplane. The purpose of these linear combinations is to “fill” the subspace described by ***X*** with as many linear combinations of input samples into ground vectors ***Y*** as needed to supply enough combinations to the projection process implied in the next step. A non-linear mapping may be introduced in Equation (5) with two purposes: to limit the values of the components of ground vectors ***Y*** to a given interval (typically [0, 1]) and to introduce some distortion in the representation space to help with non-linear separable spaces (typically sigmoid, radial basis functions or other limiting kernels may be used). This process is represented in

(6) $$\begin{array}{l} Y=f_{1}\left\{W_{1}X\right\} \end{array}$$

where ***f***_1_{·} is the non-linear mapping kernel used. In the present case a sigmoid mapping to the interval [0, 1] has been used with the purpose of easing the optimization process implied in the estimation of the output layer matrix. The third step consists in elaborating a new linear combination of the ground vectors ***Y*** given by ***z*** which is a vector intended to be as close as possible in terms of least squares to the expected target label ***t***

(7) $$\begin{array}{l} z=W_{2}Y \end{array}$$

such that fixing adequately the mapping weights ***W***_2_ the approximation error

(8) $$\begin{array}{l} e=t-z=t-W_{2}Y \end{array}$$

may be reduced to a minimum norm by an optimal estimate of the second layer mapping

(9) $$\begin{array}{l} W_{2\mathrm{op}}=\arg\left\{min\left[e^{2}\left(W_{2}\right)\right]\right\} \end{array}$$

It may be shown that ***W***_2*opt*_ can be estimated as by the following expression

(10) $$\begin{array}{l} \;W_{2\mathrm{op}}=P_{2}t;\;P_{2}=P^{T}{\left(YY^{T}\right)}^{-1} \end{array}$$

where the projection matrix ***P***_2_ is the minimum norm least squares inverse matrix (Moore-Penrose pseudoinverse) minimizing the error between the target ***t*** and the estimated label ***z*** (Barata and Hussein, 2012). This optimization problem could be solved by an iterative approximation (adaptive methods), as in classical back propagation weight adjustment, or by block estimation, as it is the case in the present study. Adaptive methods are more convenient if input samples ***X*** are expected to vary between training epochs, whereas block methods are recommended if no substantial changes are expected between training and testing conditions, and if a lower computational cost is required.

**Figure 9:**
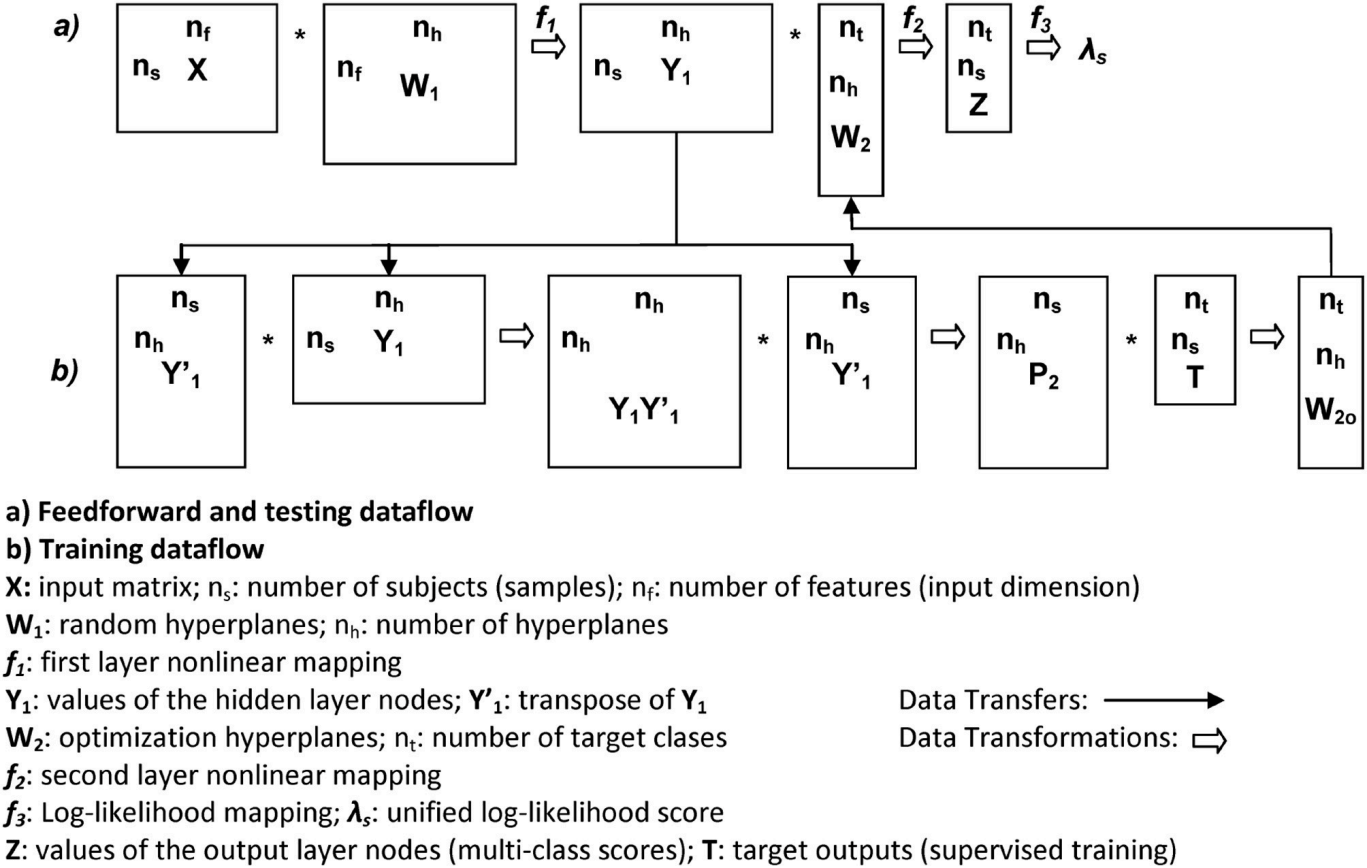
Data structures and flow of the RLSFN. Matrix dimensions are annotated close to rectangle sides (rows to vertical, columns to horizontal).

The structure of the network is depicted in Figure 9 for a better understanding of the training and testing dataflows.

Once the network has been trained, a slight modification was proposed with respect to the original formulation of the RLSFN to produce more robust classification scores. This modification consisted in redefining expression Equation (7) as

(11) $$\begin{array}{l} z=f_{2}\left\{W_{2}Y\right\} \end{array}$$

where ***f***_2_{·} is a sigmoid centered in the origin, mapping the output to the interval [0, 1]. The purpose of the sigmoid is to produce an output vector ***z*** for each sample vector ***x*** such as that the matrix ***Z*** ϵ ${\mathbb{R}}^{n_{s}\text{x}2}$ will become the output matrix, composed of *n*_*s*_ rows (the number of samples, with as many rows as subjects in ***X***, distributed as pathologicals and normatives), and two columns, whose elements *t*_*s*_ ϵ {0,1} are expected to take values tending to 0 or 1, depending on the specifications of the training matrix ***t*** ϵ ${\mathbb{R}}^{n_{sf}\text{x}2}$. In this way, each pair of values {z_*sp*_, z_*sn*_} of ***z***, as the estimated response to input sample ***x***_*s*_ from subject *s* are interpreted as the membership probability of being pathological or normative, respectively

(12) $$\begin{array}{l} z_{sp}=p\left({\text{x}}_{s}|\Gamma_{p}\right);\;z_{sn}=p\left({\text{x}}_{s}|\Gamma_{n}\right) \end{array}$$

where Γ_*p*_ and Γ_*n*_ are respectively the ideal distribution sets of AKV probability distributions from potentially pathological and normative subjects. A specific mapping can be used to estimate the normative membership for sample *s* in terms of a log-likelihood ratio (λ_*s*_) between the normative or pathological probability memberships as

(13) $$\begin{array}{l} \lambda_{s}=\text{log}\left\{\frac{p\left({\text{x}}_{s}|C_{p}\right)}{p\left({\text{x}}_{s}|C_{n}\right)}\right\}=\log z_{sp}-\log z_{sn} \end{array}$$

The value of this log-likelihood will imply labeling a sample as pathological subject when λ_*s*_> 0 and labeling it as normative otherwise. Therefore λ_*s*_ will be interpreted as a unified score be compared to targets, in which process four possibilities could be faced: a sample from a subject originally labeled as pathological, producing a score to be interpreted as pathological (true positive); a sample originally labeled as pathological, producing a score interpreted as normative (false negative); a sample originally labeled as normative, producing a score interpreted as normative (true negative), and a sample labeled as normative, producing a score interpreted as pathologic (false positive). Classically, the ratio between true positives and all the samples labeled originally as positives is known as the Sensitivity of the classifier. Similarly, the ratio between true negatives and all the samples labeled originally as negatives is known as the Specificity. The Accuracy is the sum of true positives and negatives relative to the whole sample set. The detection strategy is based in fixing a given threshold θ_*r*_ which explores the whole span from the minimum score value to the maximum (λ_*smin*_ ≤ θ_*r*_ ≤ λ_*smax*_) in a step-wise sweep, estimating the number of true positives and negatives attained at each step. Plotting the number of true positives against false negatives gives a figure of merit for the classifier, which is the Receiver-Operator-Characteristic curve (ROC). Another figure of merit is the Area Under the ROC (AUC). Log-likelihood scores have been successfully used in Biometry (Taroni et al., 2006). Among other properties, they allow to model the detection process in terms of moving thresholds plotting the number of false positives vs. false negatives in logarithmic scale, giving place to a figure of merit as a Detection-Error- Trade-off curve (DET), see Martin et al. (1997). The point where false positives and false negatives meet (or come closest together) is known as the Equal-Error-Rate (EER), and is also a figure of merit. These estimations are accompanied by the value of the optimum threshold, θ_*rop*_, corresponding to the EER point.

### Materials

Studies in monitoring PD dysarthria from speech articulation are mainly based on indices as the Vowel Space Area (VSA) and the Formant Centralization Ratios (FCR) as mentioned before, which depend on the positions of the first two formants *f*_1_ and *f*_2_ in extreme vowels (typically the trio [a:], [i:], and [u:] are used). The vowels are to be sustained, and average estimations from formant clusters are evaluated. Therefore, these indices stand on static measures of the articulator positions. The present study is intended to explore the dynamic behavior of vowel emission in open sustained vowels, as [a:] assuming that formant positions may slightly change responding to phonation instability as a consequence of the patient's inability to keep fixed articulation positions. The main difference is that the emphasis is placed in estimating these instabilities by the probability distributions of vowel kinematics, instead of searching for vowel spaces or average positions. The descriptors will be the distribution functions, instead of their second moments. Under a statistical point of view it would be expected that a more complete representation could be produced (working hypothesis). To check this hypothesis, vowel utterances from the Czech Parkinsonian Speech Database (PARCZ) were used. This database was recorded at St. Anne's University Hospital in Brno (Czech Republic). Patients were fully informed of the protocol and reach of the study, and accepted willingly to participate by signing an informed consent for their voice to be recorded and personal data to be included in an anonymous database for the study. The study and informed consent were approved by the ethical committee at St. Anne's University Hospital. The database contained recordings from four sets of five Czech vowels ([a:], [e:], [i:], [o:], and [u:]) uttered in four different ways: modal short vowels, modal long vowels, stressed long vowels and weak long vowels (soft, but not whispered). The subsets selected for the present study corresponded to phonations of long stressed [a:] at maximum loudness, from both male and female speakers, pathological and normative. The recordings were taken at 16,000 Hz and 16 bits, and segments of 500 ms were selected for analysis. A second database used for normative purposes was recorded from a set of 50 male and 50 female normative subjects free from organic or neurologic pathology selected by the ENT services of Hospital Universitario Gregorio Marañón of Madrid (HUGM). Long sustained vowels ([a:]) were recorded at a 44,100 Hz sampling frequency and 16 bits from each subject. When used in the experiments described, both the PARCZ and HUGM recordings were down-sampled to 8,000 Hz. The numbers of subjects included in the study are given in Table 2.

**Table 2:** Subjects considered in the study.

**Gender/Condition**	**Normative PARCZ**	**Normative HUGM**	**Pathological PARCZ**
Males	26	24	53
Females	25	25	38

**Figure 10:**
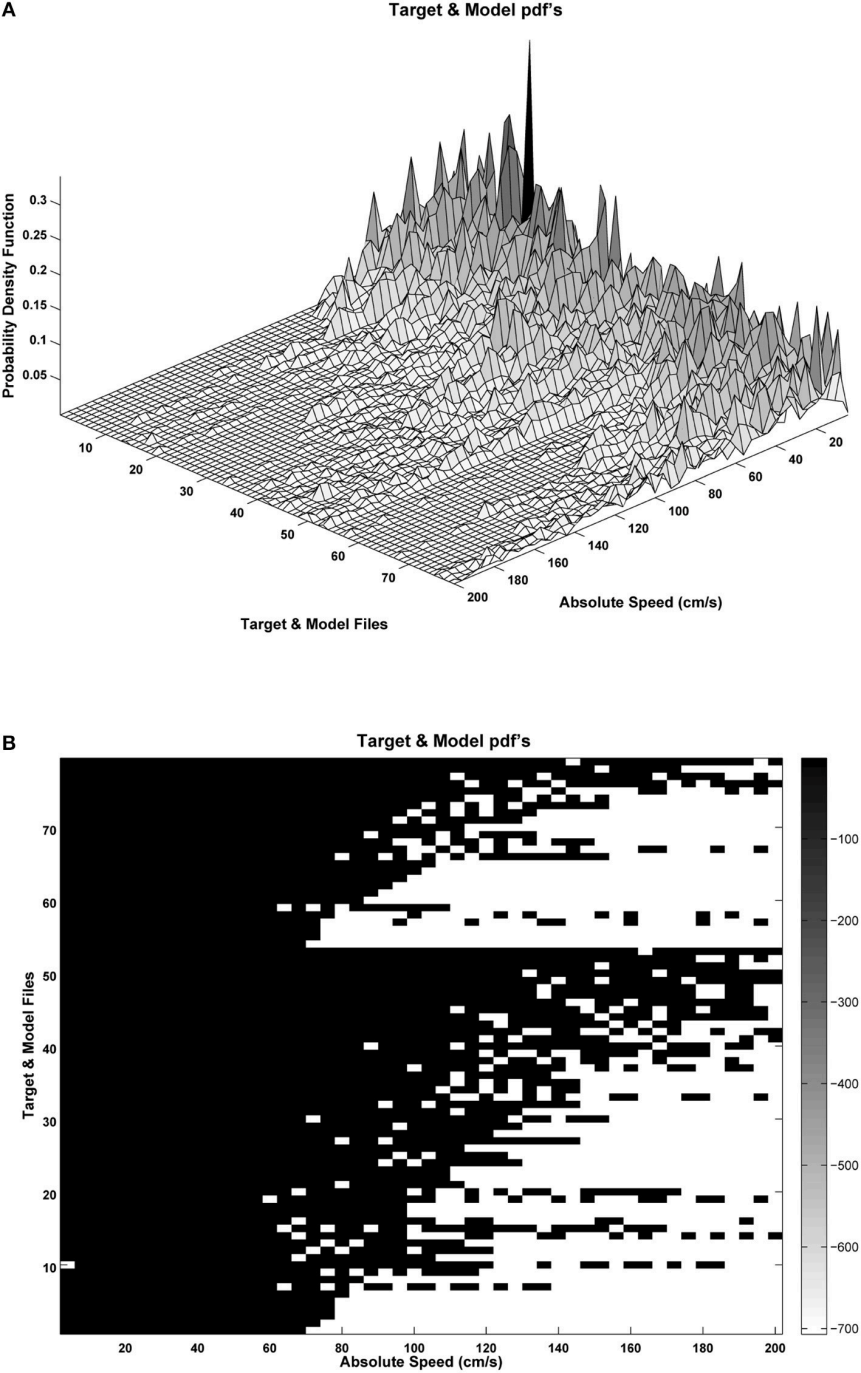
Probability distributions for the male set samples: **(A)** Target (pathological: rows 1–53) and Model (normative: rows 54–79) samples ordered by the Kulback-Leibler Divergence (KLD) between the sample to the average normative set; **(B)** silhouette of the probability distribution logarithm, showing probability density concentrations toward low or high absolute velocity values. Dispersion of the probability distribution may be induced by phonation instability as a consequence of pathological behavior.

The first two formants were estimated using inverse adaptive filtering (Gómez et al., 2009) and the respective probability distributions of the absolute kinematic velocity were calculated as by expression Equation (4). An example of the distributions for the male set (26 normative and 53 pathological) is shown in Figure 10A.

It may be seen in Figure 10B that the target sample set (rows 1–53) is ordered as a matrix, where the lowest-order rows correspond to the distributions showing less dispersion, whereas the highest-order rows include the distributions showing a larger dispersion. Similarly the model set (rows 54–79) are ordered from the less to the most disperse distributions. This matrix and a similar one for the female sample sets will be the input to the network training process following expressions Equations (5–10).

## Results and discussion

Before running detection experiments it is of vital importance to determine if the datasets to be contrasted (pathological or target vs. normative or model) show differentiate statistical distributions under classical hypothesis test conditions. Acknowledging their non-normality, and having into account that a complete analysis should contrast any distribution in the pathological dataset against any other in the normative dataset, which would generate a matrix of contrast data, difficult to interpret, a test on the average distributions has been chosen as an intermediate and more compact choice. Therefore, in Table 3 below the results of the contrastive tests are provided, in a similar way to the one to one analysis given in Table 1 for the introductory examples on the five-vowel system.

**Table 3:** Reference tests between the AKV distributions of the different PD and normative datasets.

**Dataset/Test**	**Test result**	**Test interpretation**
1. Normative males (model)–Lilliefors test	*p* < 0.001	Rejects normality
2. PD male patients (target)–Lilliefors test	*p* < 0.001	Rejects normality
3. PD vs. Normative males–Kolmogorov-Smirnov (*p* < 0.05)	0.363%	Good separability
4. Normative females (model)–Lilliefors test	*p* < 0.001	Rejects normality
5. PD female patients (target)–Lilliefors test	*p* < 0.001	Rejects normality
6. PD vs. Normative females–Kolmogorov-Smirnov (*p* < 0.05)	0.316%	Good separability
7. Joint Normatives (model)–Lilliefors test	*p* < 0.001	Rejects normality
8. Joint PD patients (target)–Lilliefors test	*p* < 0.001	Rejects normality
9. Joint PD vs. Normatives–Kolmogorov-Smirnov (*p* < 0.05)	0.302%	Good separability

The data subsets considered were normative and PD male subjects (26 and 53 subjects, respectively), normative and PD female subjects (25 and 38 subjects, respectively) and joint normative and PD subjects (51 and 91 subjects, respectively). Each data subset was first tested for normality using Lilliefors test. A *p*-value below 0.05 would reject the null hypothesis (normal distribution membership). It may be seen from tests in rows 1, 2, 4, 5, 7, and 8 that the null hypothesis was rejected for all data subsets below the lowest *p*-value tabulated (0.001), consistently with their quadratic nature (following χ^2^ distributions). The next comparison consisted in testing each distribution from each pathologic subset (males, females, and joints) vs. each distribution from each normative subject in the respective subset (males vs. males, etc.). Therefore, for the male subsets 53 × 26 = 1,378 were carried on; similarly 38 × 25 = 950 tests corresponded to the female dataset, and 91 × 51 = 4,641 tests corresponded to the joint datasets. The number of results not rejecting the null hypothesis (that both the pathological and the normative histograms came from the same distributions, therefore that the pathological and normative samples could not be differentiated) were added up and divided by the total number of tests for that subset. This figure multiplied by 100 would give the percent of cases in which the test could not differentiate between normative and pathologic. It may be seen that the Kolmogorov-Smirnov test failed in differentiating 0.363% (males), 0.316% (females), and 0.302% (joint) of the pathologic cases from the normative ones.

**Figure 11:**
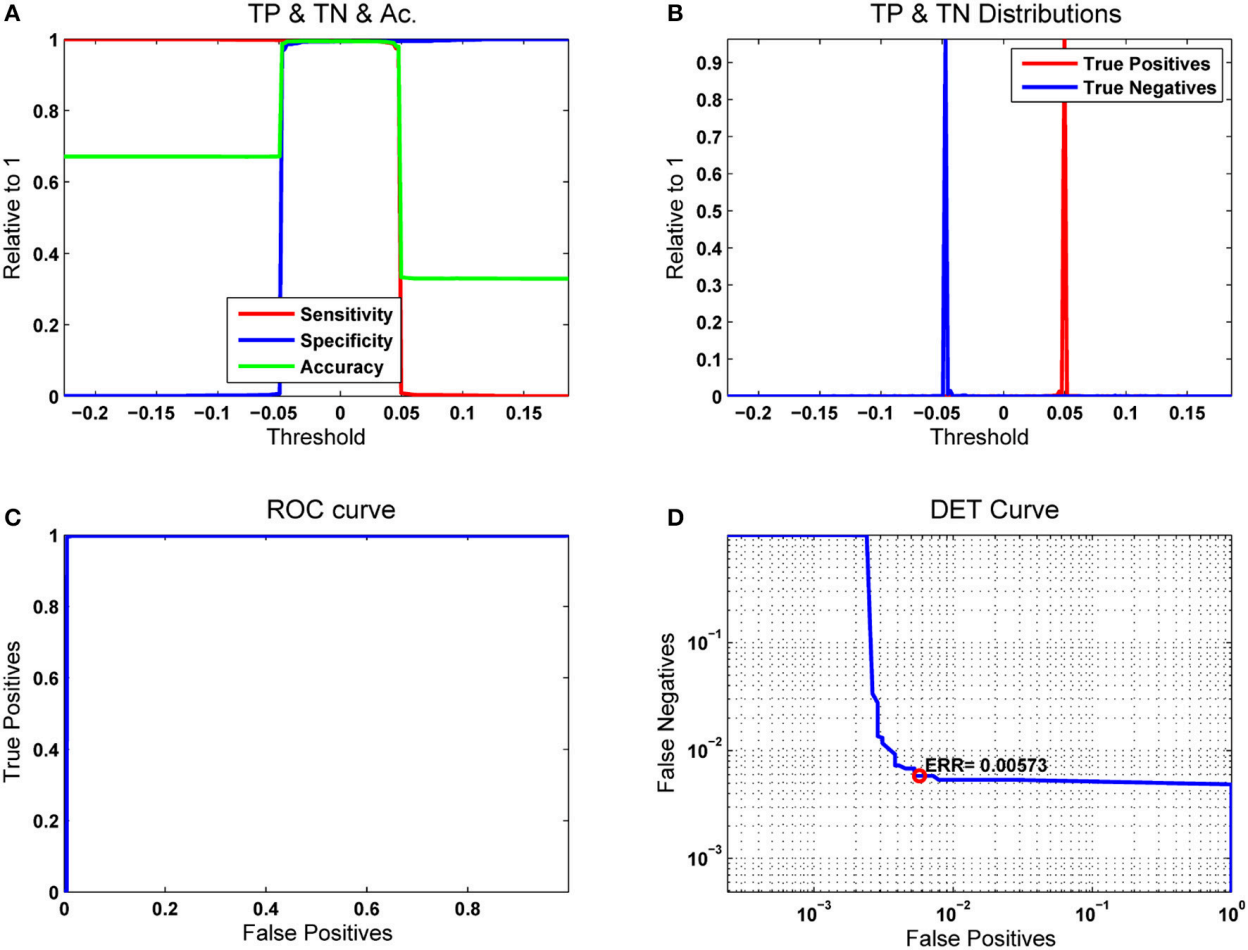
Results for a single pass of the leave-one-out strategy. The number of hyperplanes in the hidden layer was *n*_*p*_ = *200*. **(A)** Merit figures; **(B)** True-Positive and True-Negative distributions; **(C)** ROC curve; **(D)** Equal Error Rate curve.

The first classification experiment using the RLSFN consisted in running a single pass of the train and test algorithms to determine the figures of merit. The training pass used all the normative and pathological samples in each data subset (males and females) except one, which was used for testing, repeating this process over the whole sample set in a leave-one-out strategy. Figure 11 gives the results in terms of Sensitivity, Specificity, Accuracy, ROC and DET curves for the male dataset (53 pathological and 26 normative samples) from PARCZ (Table 2).

In Figure 11A the sensitivity (true positives with respect to original numbers of positives) is plotted in red as a function of the moving threshold θ_*r*_. It may be seen that for low values of θ_*r*_ the number of positives detected is 100%, but at the cost of missing all the negatives (blue line, giving the specificity as the number of true negatives with respect to the original number of negatives). Both curves cross and overlap between −0.05 ≤ θ_*r*_ ≤ 0.05. A compromise is established on the accuracy (green), which is evaluated adding the values of true positives and negatives with respect to the total number of original samples (positives and negatives). The maximum value of the accuracy is aligned with the optimal threshold θ_*rop*_. Figure 11B considers the sensitivity and specificity as the cumulative and the complementary distribution functions of the true positive and negative probability distributions. Figure 11C represents the receiver-operator-characteristic curve (ROC) in terms of the true vs. false positives, which measures the ability of a detector to distinguish between true and false alarms. The closest this curve is to the upper left corner of the diagram, the better the detector performance. This is estimated evaluating the area under the ROC curve (AUC). It is easy to see that a perfect detector would show an AUC = 1. In cases where the curve sticks tight to the ordinate axis it may be difficult to see how close it is with respect to the upper left corner. In this case, the detector-error-trade-off curve (DET) as in Figure 11D gives a more precise and meaningful view The DET curve is a plot of the complementary ROC in logarithmic scale, therefore the details of sample inclusion and exclusion from the true positive and negative sets are seen as stair steps in the bend. Another figure of merit is the equal error rate (EER), which is the point at which the numbers of false positives and negatives come to a tie. This score is also very important to fit detection conditions toward reducing the number of false negatives (crucial strategy in clinical applications) at the cost of increasing the number of false positives.

**Figure 12:**
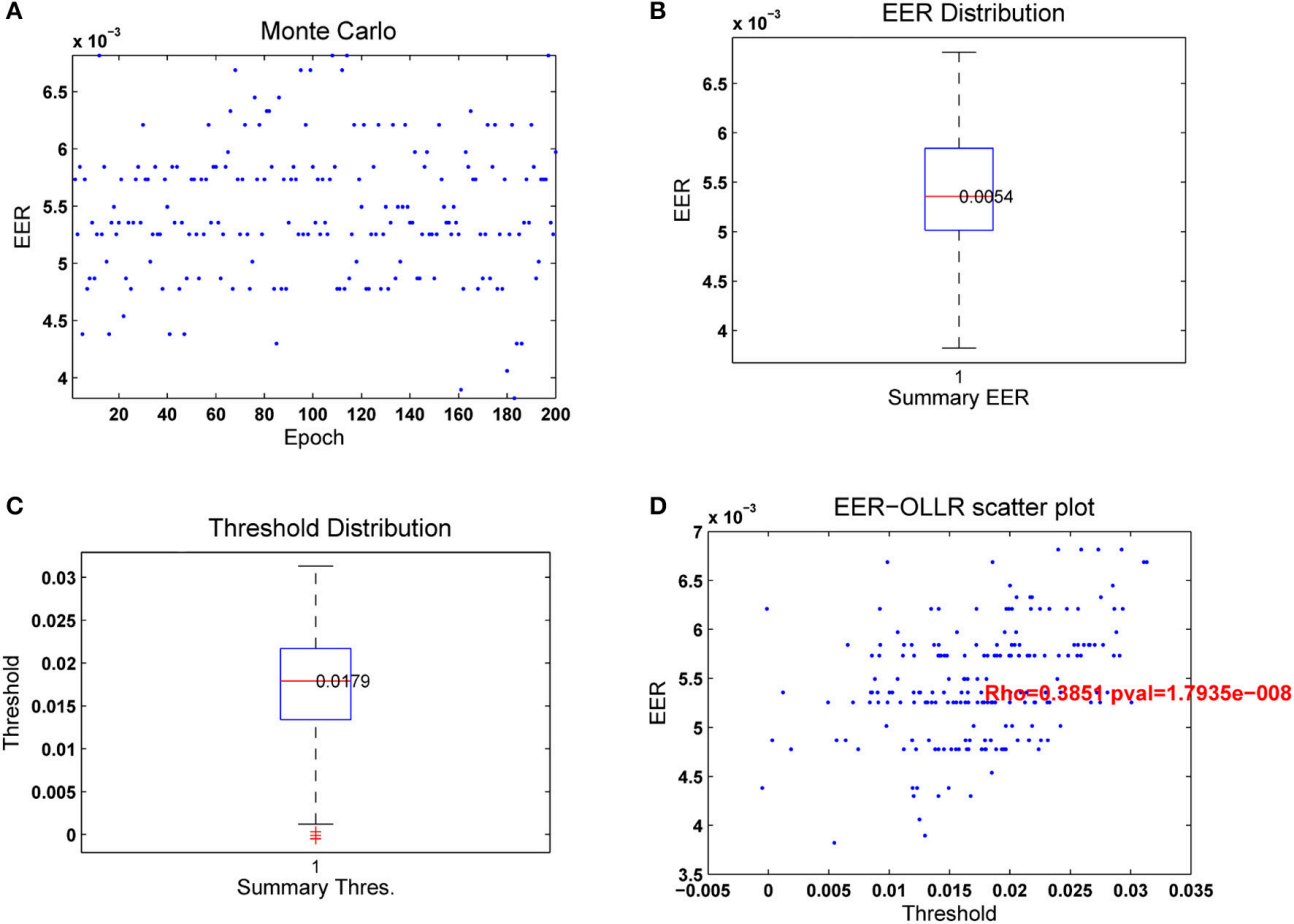
Results from 200 passes of the leave-one-out strategy. The number of hyperplanes in the hidden layer was *n*_*p*_ = *200*. **(A)** Merit figures; **(B)** True-Positive and True-Negative distributions; **(C)** ROC curve; **(D)** Equal Error Rate curve.

Due to the stochastic nature of the RLSFN, the results for matrices ***W***_1_ and ***W***_2_ will be different if different training processes are repeated. The figures of merit, given by the EER and the optimal threshold θ_*rop*_ will vary correspondingly. To understand how these scores distribute under random setting conditions, Monte Carlo simulations have been conducted. The simulations were used to obtain estimate averages of the ERR and Threshold values. Each simulation epoch was started initializing the weights of the input hyperplanes (***W***_1_) with random numbers, and following the data flow given in Figure 9 for training and testing. The results of one of these runs for *n* = *200* simulation epochs is shown in Figure 12 for the same male dataset.

In Figure 12A it may be seen that the EER tends to distribute in horizontal lines within a central band between 4.5 and 6.5·10^−3^. The apparent grouping in lines is due to the discrete nature of the classification process, as only a few samples are misclassified in each experiment, with similar results. The distribution of the EER is given in Figure 12B as a boxplot. The median is 5.4·10^−3^, with few values out of the central band. The distribution of the optimal threshold values θ_*rop*_ given in Figure 12C is also regular, the median being at λ_*s*_ = 0.0179, and the second and fourth quartiles symmetrically distributed. The number of outliers found is testimonial. Figure 12D shows the scatter plot of ε_*erop*_ vs. θ_*rop*_. The diagram shows a certain correlation, which can be estimated around ϱ = 0.3851 with a *p*-value of 1.79·10^−8^. The average detection results after 200 Monte Carlo runs of a 200 hyperplane hidden layer RLSFN are given in Table 4.

**Table 4:** Figures of merit for the male and female subsets after 200 Monte Carlo runs.

**Score/Dataset**	**Males**	**Females**
Sensitivity	0.9946	0.9942
Specificity	0.9944	0.9941
Accuracy	0.9945	0.9942
Area under the curve	0.9947	0.9945
Equal error rate	0.0054	0.0057

These results are especially relevant to fix the sub-optimal value of the detection threshold in the test phase. The stochastic behavior of the RLSFN affects the reproducibility of the results, as two different runs of the train phase will produce different results. Therefore, detection accuracy can only be estimated in its dispersion and expected values by repeating training runs. After repeating the training phase for 200 simulations, the results of the accuracy as the median, first and third quartiles (within parenthesis) are given in Table 3. The pathological dataset is respectively the male or female in PARCZ, whereas the normative datasets used are the PARCZ or the HUGM.

It may be seen that the accuracy is almost the same for both gender datasets, being slightly better for the HUGM normative dataset than for the PARCZ. As both datasets are comparable, the only reason favoring the HUGM dataset is that it consists of carefully maintained phonations of [a:] at a modal phonation, whereas PARCZ normative subjects were asked to produce phonations at a louder (stressed) modality, therefore the articulation gesture could be subject to more involuntary modifications. In any case, it may be seen that the AKV probability distributions seem to capture an important part of articulation instability to ease a clear cut between pathologic and normative articulation. The straight forward configuration of a RLSFN is capable of achieving acceptable detection ratios, which do not let much margin for further improvements. The tuning of the classifier is quite sharp, depending mostly on the number of hyper-planes used in the hidden layer. A clear decline in the EER is observed when this number (*n*_*h*_) is raised from 60 to 80. Above this figure, the EER seems to remain at a low value. Below 60, there are still many misclassifications affecting the data samples used in the training phase. Above 80, only samples from the leave-one-out set are marginally misclassified. Apparently, the ability of the pseudo-inverse projector to capture the relevant relations involving generalization is finely tuned using the least squares algorithm to estimate the pseudo-inverse using expression Equation (10). It is interesting to compare at this point the results obtained by the RLSFN with those produced by non-parametric tests, as the ones given in Table 5. It may be seen that the optimal number of misclassified samples from the male dataset is 0.54%, in the same order of magnitude than the percentage of pathological samples not rejecting the null hypothesis in the Kolmogorov-Smirnov test (0.363%), whereas these respective figures are 0.57 and 0.316% for the female subset. It seems that the classification results are well supported by Kolmogorov-Smirnov tests. As a remark, it may be mentioned that the decoding results of both the male and female data subsets are quite comparable.

**Table 5:** Average detection accuracy for the male and female datasets using the PARCZ and HUGM normative databases for 200 simulation runs.

**Gender/Accuracy (%)**	**Normative PARCZ**	**Normative HUGM**
Males	99.46 (99.36–99.51)	99.80 (99.78–99.83)
Females	99.42 (99.78–99.81)	99.81(99.78–99.87)

*The number of hyperplanes in the hidden layer was np = 200*.

Another fact to be considered is that of computational expenses. As the computation is deterministic and dataflow is predictable, computational costs are assumable, and parallelization is a clear option. Computational costs for a 200-epoch run on an i7 core at 3.4 GHz workstation running MATLAB with no special optimization improvements is of 648.97 s with an average of 3.425 s per run. Finally, as the residual improvement depends on the generalization capability of the RLSFN, further improvements could still be possible if nonlinear region-distorting mappings other than the sigmoid, as radial basis functions were used for ***f***_1_{·}. This possibility and the use of SVM's are potential continuation lines of further research.

## Conclusions

The aim of the work presented is to find out more compact, sensitive and semantic features of speech articulation by Parkinson Disease patients, which could be used in high accurate detection experiments (Cecchi, 2017). In this kind of problems the accuracy of the classifier is to be attributed to the capability of the input mapping structures to create separate and clear representations of input data, which can be associated to target scores in the output, as well as in the consistence of data. The most problematic inconsistence of data is ambiguity. As input data are produced under a pre-defined separation of samples into pathological and normative, and the feature extraction algorithms are producing estimates of the AKV distributions “blindly,” it is highly possible that some samples from pathological subjects may show a higher stability than expected. Conversely, due to articulation hesitation, for instance, a normative subject could produce unstable articulation. Separating these two types of behavior by a clear cut is always conditioned by a background group of samples where the initial hypothesis of stable articulation need not be precisely followed. The main contributions to be found in this work with respect to early work are:

- The definition of a neuromechanical model of jaw and tongue.
- The proposition of a kinematic variable with the statistical properties of a two-degree χ^2^ distribution as the classification feature.
- The modification of a RLSFN to include log-likelihood scores for optimal decision taking.
- The use of vowel-based data subsets to characterize the stability of phonation
- The use of Monte Carlo simulations for the validation of the stochastic nature of the results
- The use of non-parametric tests to avail the consistency of the data used in the experiments.

Summarizing, the following conclusions from the work presented can be derived:

- Modeling the dynamical behavior of the articulation processes by means of the AKV seems to be efficient in retaining the most important facts affecting speech stability.
- The representation of the statistical instability of speech articulation by means of AKV distributions seems to be compact enough to afford classification processes between normative and pathologic samples.
- The instability behavior is apparently well compacted in the AKV distributions to allow their classification using RLSFN's with only two layers and simple nonlinear mapping kernels.
- A log-likelihood ratio on the output complementary target output vectors can provide a meaningful score, which eventually, may be compatible with a severity degree.
- Generalization seems to behave relatively well to ensure high detection accuracy scores.
- Sensitivity, Specificity and Accuracy scores are well over 99% for both gender data subsets.
- More experimentation is needed in this sense, both enlarging the normative and pathologic databases with a larger number of subjects and with multiple sessions.
- Distinguishing other factors as aging, and the application to other other Parkynsonian syndromes, such as progressive supranuclear palsy (PSP) and multiple system atrophy (MSA). Differential diagnosis among these syndromes is a rather challenging task.

The future lines of work are to improve the research results, including more samples from different subjects, and similarly, more phonations from the same subjects to better assess intra-speaker variability and to provide results from a longitudinal study. This means processing larger databases, in which case the code should be optimized in order to reduce computational costs. The use of look-up tables to implement nonlinear mappings, and optimizing matrix inversions in Equation (10) are other key improvements to speed up calculations. Merging these articulation features with phonation ones would add up a better description to both normative and pathologic subjects. The use of nonlinear kernels in the second stages of classification could be useful in this case, as possibly data distributions would become more complex.

## Author contributions

PG: Proposing the use of kinematic variable distributions as a feature vectors for classification. JM: Development and collection of the patient database. JF: Proposition and revision of the adequate mathematical methods. DP: Selection of the adequate classifiers. AG: Algorithmic Programming and Database Processing. VR: Database Evaluation and Processing. ZG: Development and collection of the patient database. ZS: Database Collection, Backannotation and Preparation. IR: Clinical experiment design and validation. IE: Clinical inspection and validation. MK: Establish the clinical classification quality standards.

### Conflict of interest statement

The authors declare that the research was conducted in the absence of any commercial or financial relationships that could be construed as a potential conflict of interest.
